# Vaping Trends and Outcomes in Primary Total Joint Arthroplasty Patients: An Analysis of 21,341 Patients

**DOI:** 10.5435/JAAOSGlobal-D-22-00110

**Published:** 2023-01-13

**Authors:** Thomas Bieganowski, Vivek Singh, David N. Kugelman, Joshua C. Rozell, Ran Schwarzkopf, Claudette M. Lajam

**Affiliations:** From the Department of Orthopedic Surgery, NYU Langone Health, New York, NY.

## Abstract

The effect of vaping on outcomes after total joint arthroplasty (TJA) and its prevalence in this patient population remain unclear. The purpose of this study was twofold: (1) to investigate the trends of vaping in TJA patients compared with patients who underwent routine physical examination and (2) to examine the influence vaping has on outcomes after TJA.

**Methods:**

Patients were classified as never vaped, former vape users, or whether they reported current vaping (CV). TJA patients were further classified based on whether they had no exposure to tobacco or vaping (NTNV), tobacco only (TO), both tobacco and vaping (BTV), or vaping only (VO).

**Results:**

The TJA group exhibited a steady trend of patients with CV status (*P = 0.540*) while patients in the routine physical examination cohort demonstrated a significant upward trend in CV status (*P = 0.015*). Subanalysis of TJA patients revealed that those in the VO category had significantly higher mean surgical time (*P < 0.001*), length of stay (*P = 0.01*), and rates of readmission (*P = 0.001*) compared with all other subgroups.

**Conclusion:**

We found steady or increasing trends of electronic cigarette exposure in both groups over time. Additional efforts should be made to document electronic cigarette exposure for all patients.

Although there are various smoking cessation aids in use, electronic cigarettes (ECs) have gained recent popularity as a safer alternative to standard tobacco-based cigarettes.^1,2^ ECs have three different features: a power source, a heating coil, and e-liquid.^3^ The e-liquid contains a combination of solvents as well as different flavorings and nicotine, which ranges in concentration from 0 mg/mL to over 25 mg/mL.^4^ The variety of delivery methods and flavors has made use of these devices a stand-alone social habit, even among those who have never used traditional cigarettes.^5^ Several studies have examined the increasing recreational use and addictive nature of ECs, especially among teenagers.^6,7,8,9^

Widespread efforts have been made within the medical community to ensure documentation of smoking status and tobacco use.^10^ However, the same cannot be said for documentation of EC use. Unlike cigarettes, reproducible methods of quantifying exposure to ECs have not yet been developed.^11^ There are concerns that vaping has become an epidemic in its own right, but long-term outcome data to support this claim are limited.^12,13^ Specifically, little is known about the effects of vaping on outcomes after total joint arthroplasty (TJA). This lack of published evidence precludes arthroplasty surgeons from making informed decisions on whether to include EC exposure in their perioperative optimization history and strategy. Optimization decisions can have a profound effect on recovery and also correlate with sustained behavioral change long after surgery.^14,15^ Lack of awareness of vaping habits during preoperative optimization may represent a missed opportunity to influence TJA outcomes and overall patient well-being.

To the best of our knowledge, no previous study has outlined the trends of EC use in orthopaedic patients compared with the general public. Therefore, the purpose of this study was to gauge the prevalence of documented EC use in TJA patients and general medical patients. Increased awareness of vaping as an independent social habit will allow more accurate documentation so that the effect of EC exposure on surgical and other outcomes may be extrapolated.

## Methods

### Study Design

An IRB-approved retrospective analysis of prospectively collected patient data from a single, urban, high-volume academic medical center was conducted. Patients were stratified into two cohorts: (1) those who underwent primary TJA at our institution between January 2013 and December 2021 and (2) all patients who had a routine physical examination (RPE) during the same period. Patients were then subcategorized based on documentation of EC use. Classification included never vaped (NV), former vape (FV) users, or currently vaping (CV). To facilitate analysis of outcomes, TJA patients were further classified based on whether they had no exposure to tobacco or vaping, tobacco only, both tobacco and vaping, or vaping only (VO). Using the Current Procedural Terminology codes 99385, 99386, 99387, 99395, 99396, and 99397, we were able to identify patients who underwent RPE. We noted whether EC status was not specified (left blank) in the electronic medical records. All patients 18 years or older who underwent unilateral elective primary total knee arthroplasty, primary total hip arthroplasty, or an RPE were included. Patients who underwent a primary TJA for a nonelective reason; bilateral TJA; revision TJA patients; patients with incomplete or unspecified EC status, demographic data, or outcomes data; and those younger than 18 years were excluded.

### Data Collection

Demographic variables included in our analysis were race, ethnicity, age, sex, and body mass index (BMI). In addition to demographic data, information on primary TJA procedure, RPE, and exposure to ECs was recorded. Perioperative data were collected for all TJA patients involving surgical time (minutes), length of stay (LOS, days), discharge disposition, readmission rates, and revision rates. All data were obtained using our large electronic medical records database (Epic Caboodle. version 15; Verona, WI) and deidentified on encrypted Microsoft Excel software.

### Outcome Measures

The primary outcome measures included were percentages of patients with NV, FV, or CV status. The rates of NV, FV, and CV status were collected through documentation of patient-reported exposure to ECs. Exposures are recorded in the electronic medical record and were obtained for all office visits or TJA cases where patients were queried regarding their status.

The secondary outcomes included trends of EC current use (CV status) for all patients undergoing a TJA or an RPE. The percentages of patients currently using ECs from each year were used to calculate a linear regression that represented the trend of patients with CV status over time.

In addition, a subanalysis of TJA patients was conducted accounting for both tobacco and EC exposures. The outcomes measured included surgical time, in-hospital LOS, discharge disposition, and postoperative adverse events such as readmission and revision rates. Surgical time was derived from calculating the time difference between the initial skin incision and the completion of skin closure. Length of stay was calculated by taking the difference in time between the admission date and the discharge date.

### Statistical Analysis

Statistical analyses were performed using SPSS v25 (IBM Corporation, Armonk, New York). A binary variable was created to differentiate patients who received a TJA from patients who underwent RPE. The baseline demographic and clinical characteristics used to describe our study participants were reported as means with standard deviations for continuous variables and frequencies with percentages for categorical variables. Statistical differences between continuous demographic variables were detected using independent sample two-sided Student *t*-tests. Categorical demographic variables and proportion of vape exposure during each year were analyzed with chi square (χ2) tests. Linear regression analysis was then used to determine the significance of exposure each year while controlling for all potential confounding variables. The trend over time of patients with CV status was also calculated using linear regression and reported as an unstandardized beta. A notable unstandardized beta indicates an increasing or decreasing trend while a lack of significance indicates a stable trend. All TJA patients were further stratified based on their exposure to tobacco and ECs. Univariate linear regression was used to determine the statistical significance of perioperative outcomes while controlling for potential confounding variables. Confounding variables included all statistically significant demographic variables, which involved race, ethnicity, age, sex, and BMI. A *P*-value of less than 0.05 was considered to be statistically significant for all measures used in this study.

## Results

### Patient Population

Of the total number of TJA and RPE patients identified, 3122 and 248,153, respectively, had incomplete or unspecified EC documentation, leaving 21,341 in the TJA cohort and 1,060,898 in the RPE group. Patients in the TJA cohort had a significantly higher percentage of Black or African American patients (TJA: 15.9% vs RPE: 12.8%; *P < 0.001*) and patients of Spanish or Hispanic origin (TJA: 3.3% vs RPE: 1.3%; *P < 0.001*)*.* Patients in the TJA cohort were significantly older than those in the RPE group (TJA: 64.64 ± 10.67 vs RPE: 44.81 ± 15.08; *P < 0.001*). The TJA cohort had a significantly lower percentage of women (63.0%) than the RPE cohort (66.9%; *P < 0.001*). Patients undergoing TJA had significantly higher mean BMI than those in the RPE group (TJA: 30.86 ± 6.54 vs RPE: 27.38 ± 21.00; *P < 0.001*). A full comparison of demographic variables is outlined in Table 1.

**Table 1:** Demographic Comparison

	RPE (n = 1,060,898)	TJA (n = 21,341)	*P* value
Race			<0.001
White	614954 (58.0%)	13775 (64.5%)	
Black or African American	136413 (12.8%)	3389 (15.9%)	
Asian	79261 (7.5%)	741 (3.5%)	
Other	230270 (21.7%)	3436 (16.1%)	
Ethnicity			<0.001
Not of Spanish/Hispanic origin	126870 (12.0%)	5597 (26.2%)	
Spanish/Hispanic origin	14275 (1.3%)	709 (3.3%)	
Unknown	919753 (86.7%)	15035 (70.5%)	
Age (± SD), yr	44.81 ± 15.08	64.64 ± 10.67	<0.001
Sex			<0.001
Male	351262 (33.1%)	7897 (37.0%)	
Female	709636 (66.9%)	13444 (63.0%)	
BMI (± SD), kg/m^2^	27.38 ± 21.00	30.86 ± 6.54	<0.001

BMI = body mass index, TJA = total joint arthroplasty

### Vaping Trends

**Figure 1:**
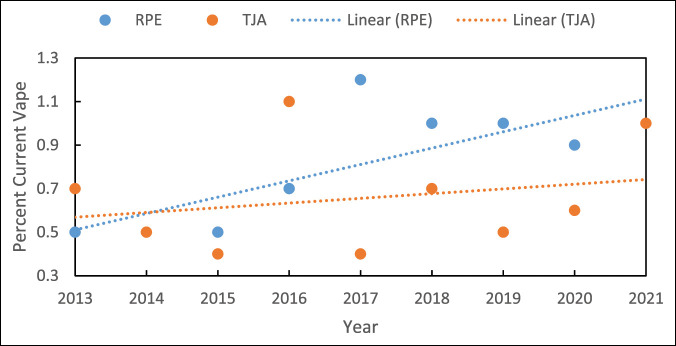
Graph showing trends in current vape status for RPE and TJA patients over time. TJA = total joint arthroplasty, RPE = routine physical examination

Documented EC exposure for both cohorts was uncommon, with an average for all patients of 3.0% overall—ranging from 2.6% to 4.1% in the TJA cohort and 2.1% to 3.5% in patients undergoing an RPE. After controlling for significant demographic differences including age, we found that the TJA cohort had a significantly higher percentage of patients with CV status in 2016 (TJA: 1.1% vs RPE: 0.7%; *P = 0.012*) while RPE patients demonstrated a significantly higher rate of CV status in 2017 (TJA: 0.4% vs RPE: 1.2%; *P < 0.001*) (Table 2). Patients in the TJA cohort had a higher percentage of former EC users (FV status) at all time points. There was a significant increase in the prevalence of CV status for the RPE group (unstandardized β: 0.075, 95% CI: 0.019-0.131; *P = 0.015*). A complete analysis of trends data is presented in Table 3 and Figure 1.

**Table 2:** Vaping Status

		# RPE Patients	% RPE Patients	# TJA Patients	% TJA Patients	*P* value
2013	Vaping status					0.556
	Never vape	13903	98.0	1071	96.9	
	Former vape	223	1.	26	2.4	
	Current vape	67	0.5	8	0.7	
2014	Vaping status					0.599
	Never vape	22025	97.8	1541	97.3	
	Former vape	403	1.8	35	2.2	
	Current vape	102	0.5	8	0.5	
2015	Vaping status					0.396
	Never vape	33911	97.7	2083	97.4	
	Former vape	634	1.8	47	2.2	
	Current vape	173	0.5	9	0.4	
2016	Vaping status					0.012
	Never vape	50436	97.3	2258	95.9	
	Former vape	1026	2.0	71	3.0	
	Current vape	383	0.7	25	1.1	
2017	Vaping status					<0.001
	Never vape	117472	96.5	2841	96.6	
	Former vape	2808	2.3	87	3.0	
	Current vape	1482	1.2	12	0.4	
2018	Vaping status					0.394
	Never vape	177415	97.0	3038	96.3	
	Former vape	3701	2.0	96	3.0	
	Current vape	1790	1.0	22	0.7	
2019	Vaping status					0.255
	Never vape	178690	97.0	2764	96.5	
	Former vape	3766	2.0	84	2.9	
	Current vape	1781	1.0	15	0.5	
2020	Vaping status					0.136
	Never vape	176778	97.0	2236	96.7	
	Former vape	3736	2.1	63	2.7	
	Current vape	1656	0.9	13	0.6	
2021	Vaping status					0.655
	Never vape	258607	97.0	2791	96.6	
	Former vape	5343	2.0	68	2.4	
	Current vape	2587	1.0	29	1.0	

RPE = routine physical examination, TJA = total joint arthroplasty

**Table 3:** Trends in Current Vape Status

	Unstandardized Beta	Pearson R	95% CI	*P*
RPE	0.075	0.593	0.019 to 0.131	0.015
TJA	0.022	0.056	−0.058 to 0.101	0.540

RPE = routine physical examination, TJA = total joint arthroplasty

### Vaping Outcomes

Of the 21,024 patients within the TJA cohort who had documented tobacco and vape status, 11,459 patients had used neither tobacco nor ECs; 8857 patients used tobacco only; 664 had both tobacco and EC use; and 44 patients were documented as VO use. After controlling for demographic differences, patients within the VO group had significantly longer surgical times (110.36 ± 41.59 minutes; *P < 0.001*) and LOS (2.34 ± 3.73 days; *P = 0.01*) compared with all other cohorts. Patients in the VO cohort also had significantly higher rates of readmission (9.1%, *P = 0.001*) compared with other subgroups. Of the 627 revisions in our initial TJA group, 63 (10.0%) were not included in the final analysis because of incomplete or absent EC documentation. The small sample size precludes meaningful statistical analysis. A comparison of all outcomes data is presented in Table 4.

**Table 4:** Outcomes

	NTNV (n = 11,459)	TO (n = 8857)	BTV (n = 664)	VO (n = 44)	*P* value
Surgical time (± SD), min	97.84 ± 31.00	97.65 ± 30.39	103.04 ± 31.71	110.36 ± 41.59	<0.001
LOS (± SD), d	2.09 ± 1.76	2.09 ± 1.61	2.19 ± 1.49	2.34 ± 3.73	0.010
Discharge disposition					0.648
Home	88.2%	89.0%	88.3%	84.1%	
Facility	11.8%	11.0%	11.7%	15.9%	
Readmission rate	3.2%	4.2%	4.5%	9.1%	0.001
Revision rate	2.4%	3.0%	2.6%	4.5%	0.060

BTV = both tobacco and vaping, NTNV = no exposure to tobacco or vaping, TO = tobacco only, VO = vaping only

## Discussion

While documentation of cigarette smoking has become a routine part of most medical visits, our findings suggest that EC documentation is missing from the health record for 12.8% of TJA patients and 19.0% of RPE patients. For those with EC documentation, a higher prevalence of use fluctuated between groups over the study period. Despite this, a statistically significant upward trend in EC users within our RPE patients cannot be ignored. To the best of our knowledge, there are no studies to date examining trends in EC use among TJA patients compared with the general population. This comparative analysis demonstrates trends of EC use within the general population that are not yet reflected in TJA patients, possibly due to their older age. This may indicate that EC use is a stand-alone social habit that selectively affects younger patients. As the RPE population ages, the proportion of EC users will require increased attention as they become candidates for TJA.

Our study revealed a steady trend in CV status for patients undergoing TJA and a significantly increasing trend of CV status in RPE patients (*P = 0.015*). These results support previous literature that has raised concerns over the prevalence of vaping as an independent habit, particularly among younger people.^7^ Sun et al.^16^ created a new metric entitled “nicotine product days” and found a notable increase in the measure from 2017 through 2020, which they attributed to the growing popularity of ECs. Obisesan et al.^8^ examined a national behavioral risk surveillance database to determine the degree of EC use nationwide and found a steady increase in EC exposure over three years beginning in 2016 and ending in 2018. Cho et al. examined the marketing of ECs from 2017 to 2019 across multiple countries and found that adolescents in America had a markedly higher prevalence of frequent exposure to EC advertising (31.7%) than adolescents in Canada (24.2%) and England (25.2%; *P* < 0.001).^17^ Both the study by Cho and this study represent an important cultural shift in EC use as a stand-alone social habit.

Although there is evidence that ECs expose users to lower levels of nicotine than tobacco-based cigarettes, the adverse health effects of EC use are not well-characterized in the medical literature. The Royal College of Physicians recently asserted that the long-term effects of EC vapor are <5% as harmful as that of tobacco smoke.^18^ Similarly, the US National Academies of Science, Engineering, and Medicine concluded that vaping is less hazardous than cigarette smoke.^18^ Despite this, there are several reports outlining the detrimental effects of vaping, many of which may influence outcomes after orthopaedic surgery. Clapp et al. indicated that some of the flavorings used in ECs may blunt the immune response^19^ while Page et al.^20^ reported on decreased cutaneous blood flow immediately after EC exposure. Both of these factors influence recovery and wound healing after TJA.

Our results indicate that TJA patients who were exclusively exposed to EC vapor had markedly longer surgical times and LOS as well as markedly higher rates of readmission versus all other TJA patients, including those who smoked traditional cigarettes. Although there were relatively few patients in this group (n = 44), these outcomes should raise concern for TJA patients who use ECs. Compared with traditional cigarettes (6.17 to 28.86 mg)^21^ or nicotine replacement therapy (5 to 52.5 mg),^22^ ECs are found to deliver less predictable quantities of nicotine (0 to 20 mg/mL),^4^ which vary by product and may change on each inhalation.^23^ ECs are often used as an augment to traditional tobacco cessation aids; however, the combination of ECs with other nicotine delivery methods could result in inadvertently high levels of serum nicotine. The influence of such potential exposures on bone physiology has been widely examined. Walker et al.^24^ described the bimodal effect nicotine may have on human bone cells whereby higher concentrations triggered cell death. Similarly, Marinucci et al. reported on nicotine-induced osteoblast apoptosis through the accumulation of reactive oxygen species.^25^ Nicholson et al.^26^ suggested that high concentrations of nicotine found within some ECs could lead to the impaired function and viability of both osteoblasts and osteoclasts. These findings may offer insight into potential challenges the surgeon may face intraoperatively. Given the lack of consistency of nicotine delivery by ECs, surgeons must consider the wide range of nicotine replacement therapies available to patients and document their use to better inform optimization strategies moving forward.

Many previous studies have demonstrated the influence of surgical procedures on behavioral habits.^27^ Hall et al.^28^ surveyed TJA patients who smoked and found that 75% quit smoking as part of surgical optimization. At a mean follow-up time of 52 months, 23% maintained abstinence, representing a much higher number than the national average. Hart et al. noted that at the eight-year follow-up, 45% of patients maintained their smoking abstinence if they quit before undergoing TJA.^29^ In addition, Hart et al. reported that serum cotinine testing before surgery significantly increased the rate of smoking cessation at the time of surgery, from 15.8% in the untested group to 28.2% in the tested group (*P = 0.005*).^30^ Our results show that despite a notable upward trend in those with CV status for patients within a general medical population, CV status in TJA patients paradoxically did not parallel this trend and, in fact, showed no notable change over time. This may represent a lag in EC use for older TJA patients, secondary to the younger age of those who use EC exclusively. We also noted that more than one in 10 TJA patients had missing or inadequate documentation of their EC exposure. Given our findings on how EC use may influence outcomes after TJA, a greater effort may be required on the part of orthopaedic surgeons to query preoperative patients about all forms of nicotine exposure. As the population ages in the United States, trends seen in our RPE population may translate to the TJA population. Therefore, it will become increasingly important to document the use of EC in the TJA population. This will allow more powerful analysis of the effect on TJA outcomes going forward and may encourage more nuanced optimization strategies.

## Limitations

This study is not without some inherent limitations. The retrospective collection of data exposes the results to bias, which we attempted to control for when factoring confounding variables into the analysis. We represented the general population based on patients undergoing RPE, but the use of this metric may influence the results. Those who choose to undergo RPE may self-select toward those more vigilant about monitoring and management of health conditions. Young, healthy patients may be less likely to undergo RPE and would thus be less likely to be included in our analysis. Those who are uninsured or have lower health literacy may have a different prevalence of EC use than a typical RPE population and may not have been included in this analysis. Our study focused only on patients presenting to a large urban academic institute. There may be regional differences in EC use in both TJA and RPE groups. Larger database studies could elucidate the generalizability of our results and highlight national trends. In addition, our study relies on documentation in the medical record to categorize EC exposure and is subject to reporting bias. The heterogeneity of available EC products makes it difficult to quantify the level of exposure. Limitations notwithstanding, our study design used a reliable and reproducible statistical methodology that provided a comparison of documented EC exposure between patients undergoing TJA and the general population over time.

## Conclusion

Statistically significant differences were observed in surgical time, LOS, and readmission for TJA patients who were exclusively exposed to ECs. With EC use on the rise in the United States, the need to identify and document patient use is increasingly important. Despite inconsistent documentation, exposure to vaping by patients at our institution has demonstrated stable or increasing trends for the younger general patient population and TJA patients. As these younger RPE patients age into being candidates for TJA, it will become increasingly important to document EC use. While the health risks of vaping are poorly understood, the increased popularity of EC use as a distinct social habit warrants consistent documentation of use by health practitioners. Only then can we accurately assess the effect of EC use on surgical outcomes and overall health. Improved recognition of ECs as a social habit can improve shared decision making for elective TJA.
